# Mindfulness and psychotic-like experiences in nonclinical populations: a systematic review and two meta-analyses

**DOI:** 10.1017/S0033291725103061

**Published:** 2026-02-04

**Authors:** Katrina Mysko, Elise Quarterman Gear, Lyn Ellett

**Affiliations:** University of Southampton, UK

**Keywords:** meta-analysis, mindfulness, nonclinical populations, psychotic-like experiences, systematic review

## Abstract

This systematic review and meta-analyses provide the first synthesis of the literature on trait mindfulness and psychotic-like experiences (PLEs). Theoretical models suggest a protective function of mindfulness and it is important to understand any potential role of mindfulness in the prevention and treatment of PLEs. We examined the following: (1) What is the relationship between trait mindfulness and PLEs in nonclinical populations?; and (2) What is the effect of mindfulness-based interventions (MBIs) on PLEs in nonclinical populations? Five databases were searched, and effect sizes were extracted for each study. Seventeen papers were included in the review. Eleven papers explored the relationship between mindfulness and PLEs, and the meta-regression found a small negative association between PLEs and mindfulness (*k* = 8; pooled correlation *r* = −0.25; 95% confidence interval [CI]: −0.37, −0.13, *p* < .001). Eight studies investigated the effect of MBIs on PLEs and the summary effect was not significant in the meta-analysis (*k* = 5; pooled standard mean difference = .09; 95% CI: −0.61, 0.79, *p* = 0.80). Overall, the findings suggest that higher levels of mindfulness are associated with reduced PLEs, with no evidence for the effectiveness of MBIs in reducing PLEs. Findings should be interpreted cautiously given the small number of studies and high heterogeneity in the meta-analyses. Future studies are needed to determine whether MBIs might prevent the transition to psychosis or an at-risk mental state and might usefully measure a broader range of clinically relevant outcomes.

## Introduction

Psychotic-like experiences (PLEs) can refer to a broad and variably defined set of subclinical or subthreshold phenomena that resemble experiences commonly associated with psychosis, such as hallucinations, delusions, and paranoid thoughts (Bourgin et al., 2020). PLEs are observed in both clinical and nonclinical populations and are understood to exist on a continuum, with varying degrees of frequency, intensity, and associated distress (Linscott & van Os, 2013; Yung et al., 2009). However, there is currently no universally accepted definition, likely because PLEs are not defined diagnostically due to their lower severity and impact on daily functioning, and shorter duration (Logoń, Świrkosz, & Kowalski, 2025). PLEs can refer to both a collective group of symptoms, such as persecutory thoughts, bizarre beliefs, and perceptual abnormalities, or single symptoms, such as paranoia *or* hallucinations. PLEs are often assessed through self-report measures, which may focus on single symptoms, such as the Peters *et al. Delusion Inventory* (Peters, Joseph, Day, & Garety, 2004), or groups of symptoms, such as the Community Assessment of Psychic Experiences (Konings et al., 2006; Villacura-Herrera, Pérez, Jones, & Núñez, 2024).

Research indicates that up to 30% of the general population report experiencing at least one PLE (Bourgin et al., 2020; Rep et al., 2023). The presence of PLEs has been associated with an increased risk of developing psychotic disorders, particularly when the PLEs are persistent and reoccurring (Dominguez et al., 2009), occur alongside anxiety (Isaksson et al., 2022), or are accompanied by substance use (Mackie, Castellanos-Ryan, & Conrod, 2011). Furthermore, PLEs have been associated with distress, depression, self-injurious behaviors, suicide attempts, and suicide deaths (Logoń et al., 2025). Consequently, understanding PLEs in nonclinical populations and identifying strategies to minimize their impact is crucial.

Mindfulness-based interventions (MBIs) have been used to reduce PLEs in nonclinical populations. Mindfulness, defined as the intentional, nonjudgmental awareness of the present moment (Kabat-Zinn, 2000), has been found to be inversely correlated with nonclinical experiences of paranoia (Pagnini, Bercovitz, & Phillips, 2018), hallucinations (Moran, Larsson, & McHugh, 2021), delusions (Oliver, McLachlan, Jose, & Peters, 2012), and overall experiences of PLEs (Torok & Keri, 2022). MBIs are therapeutic approaches that incorporate mindfulness practices, such as meditation, breathing exercises, and mindful awareness of thoughts, emotions, and physical sensations, to promote mental health and well-being (Kabat-Zinn & Hanh, 2013). The delivery of MBIs can be highly variable, ranging from face-to-face groups with 2-h weekly sessions over 8 weeks (Segal, Williams, & Teasdale, 2002) to online formats with daily 10-min audio sessions for 2 weeks (Shore et al., 2018). Mindfulness has also been incorporated into other forms of psychological support, such as Resilience Training (Burke et al., 2020) and Nature Connectedness (Muneghina, Van Gordon, Barrows, & Richardson, 2021). MBIs have been used in nonclinical populations with the aim of reducing paranoid experiences (Shore et al., 2018) and delusions (Burke et al., 2020), as well as clinical populations with participants with a diagnosis of a schizophrenia spectrum disorder (Chadwick et al., 2016; Ellett, 2013; Ellett et al., 2020; see Ellett, 2024, for a summary of published meta-analyses).

While individual studies have examined the effects of mindfulness on single PLEs (Shore et al., 2018), or general PLEs (Peters et al., 2016), there is currently no comprehensive synthesis of the impact of MBIs on PLEs. Therefore, this systematic review aimed to synthesize the existing literature examining mindfulness and PLEs in nonclinical populations. This is particularly important, as (online) MBIs could offer an early intervention that has the potential to be low cost and accessible for individuals experiencing PLEs, and may have the potential to reduce transition to psychosis. The review will address the following preregistered research questions:

1. What is the relationship between mindfulness and PLEs in nonclinical populations?
2. What is the effect of MBIs on PLEs in nonclinical populations?

## Methods

This systematic review was performed in compliance with the Page et al. (2021) Preferred Reporting Items for Systematic Reviews and Meta-Analyses (PRISMA) and was preregistered on PROSPERO on February 7, 2025 (available from https://www.crd.york.ac.uk/PROSPERO/view/CRD420250649252). The initial search took place in February 2025, and the searches were rerun in April 2025, with no new papers identified. Rayyan (Ouzzani, Hammady, Fedorowicz, & Elmagarmid, 2016) software was used to facilitate efficient organization and categorization of papers, as well as enabling a ‘blind mode’ for the independent review process.

### Inclusion and exclusion criteria

To be included in the systematic review, papers had to meet the following inclusion criteria: (1) use of cross-sectional, longitudinal, quasi-experimental, or experimental designs, including studies without a control group, waitlist control group, passive control, or active control groups; (2) adults aged 18 years or older in a nonclinical population, which was defined as participants without an active mental health diagnosis and not receiving current support for their mental health; (3) collected data of individual PLEs or general PLEs using a self-report measure and/or a mindfulness measure; (4) studies that examined the relationship between mindfulness and PLEs or studies that included either an MBI or where the intervention included a primary focus on mindfulness, defined as mindfulness present in more than 50% of the intervention sessions; (5) published in a peer reviewed journal; and (6) published in the English language.

The exclusion criteria were: (1) case studies, case series, and systematic reviews; (2) studies that included participants <18 years of age, or mixed adult and child samples, or a targeted population based on existing or previous mental health conditions; (3) unpublished dissertations and conference abstracts; (4) a primary focus on personality disorders, including schizotypal and paranoid personality disorder as the focus was on nonclinical samples.

### Database and search strategies

Five electronic databases were searched to find eligible papers: PsycINFO, CINAHL, and MEDLINE, all accessed via EBSCOhost, as well as Web of Science Core Collection and ProQuest. The following search terms were used: (“mindfulness-based interventions” OR “mindfulness based interventions” OR mindfulness OR “mindfulness meditation” OR mediatat*) AND (“non-clinical population” OR “general population” OR “sub-clinical” OR student) AND (“psychotic-like experiences” OR PLEs OR “non-clinical psychosis” OR “psychosis-like experiences” OR hallucination* OR delusion* OR schizotyp* OR psychotic OR “psychosis proneness” OR paranoi* OR persecutory* OR grandiose* OR “unusual experiences” OR “voice hearing” OR “non-clinical paranoia”). No publication date limits were set, but a filter of the English language was used.

### Screening process

**Figure 1.**
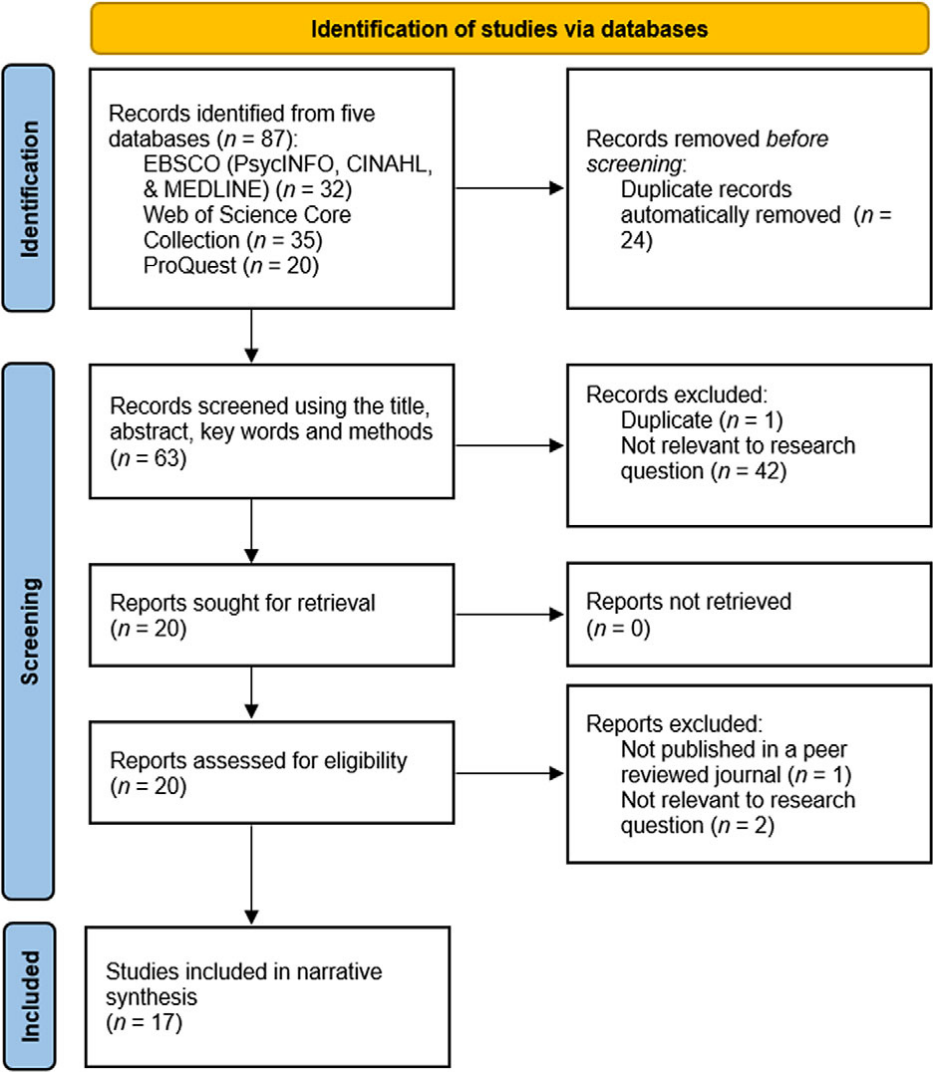
PRISMA flowchart.

Papers were initially screened using their title, abstract, keywords, and methods. Papers included at the initial screening stage progressed to full-text screening. An independent second reviewer assessed 100% of papers identified by the searches, with discrepancies between reviewers resolved by discussion. A PRISMA flowchart of paper identification and screening processes is displayed in Figure 1.

### Quality assessment

Each paper was assessed for methodological quality using the Standard Quality Assessment Criteria for Evaluating Primary Research Papers from a Variety of Fields (Kmet, Cook, & Lee, 2004). The tool consisted of 14 questions with each item scored on a 3-point Likert scale (no = 0, partial = 1, yes = 2), although four items were not used for cross-sectional and quasi-experimental studies (questions 5, 6, 7, and 12), and one item was not used for randomized controlled trials (RCTs) (question 7). The tool included guidelines for responses to each item and an equation to calculate the summary score. The following cutoffs were used for the summary score: Strong >0.80; Good = 0.70–0.80%; Adequate = 0.50–0.69; Limited <50% (Lee, Packer, Tang, & Girdler, 2008). An independent second reviewer quality-assessed four papers (22%), with discrepancies between reviewers resolved by discussion.

### Data extraction, synthesis, and meta-analysis

The key characteristics of each study and its population, as well as data related to the research questions, including effect sizes (either Pearson’s *r* or Cohen’s *d*), were extracted manually by the first author. Authors were contacted to request any missing data. For the purposes of this review, only baseline and immediate post-intervention data were extracted. Meta-analyses were planned if there were two or more appropriate papers addressing each research question, supplemented by the use of narrative synthesis. Meta-analyses were carried out using Comprehensive Meta Analysis Version 4 (Borenstein, Hedges, Higgins, & Rothstein, 2022).

A random-effects model was used for all meta-analyses to allow for substantial heterogeneity across studies, as a range of different measures were used to capture PLEs and mindfulness. Additionally, this approach was appropriate, as it was assumed there was a variation in effect sizes across the studies rather than an assumption of a single true effect size (Riley, Higgins, & Deeks, 2011). For studies exploring the relationship between mindfulness and PLEs in nonclinical populations, a meta-regression was conducted using Pearson’s correlation coefficient (*r*) of total mindfulness score and PLEs and the sample size. For studies exploring the effect of MBIs on PLEs in nonclinical populations, a meta-analysis was conducted using the sample size and means and standard deviations for the PLE measure for both the control group and experimental group at post-intervention. Statistical heterogeneity was assessed by the *Q* test and *I* ^2^ statistic. High heterogeneity was considered present if the *Q* test result was significant (*p* < 0.05) or if the *I* ^2^ value exceeded 50% (Deeks et al., 2024). To evaluate publication bias, funnel plots were visually inspected, and Egger’s test was performed. A significant result from Egger’s test (*p* < 0.05) or data points falling outside the funnel plot indicated the presence of publication bias (Page, Higgins, & Sterne, 2024). Effect sizes of Cohen’s *d* were interpreted as small (0.2), medium (0.5), and large (0.8), and effect sizes of Pearson’s *r* were interpreted as small (0.1), medium (0.3), and large (0.5), in line with conventional categorization (Cohen, 1992).

## Results

A total of 87 papers were identified and screened, 20 papers were extracted and assessed for eligibility, and 17 papers were eligible for inclusion and were included in the final review. One paper contained three studies, two of which met eligibility criteria and were treated as two separate studies. See Table 1 for a summary of study characteristics for the papers included in the review.

**Table 1.** Summary table of the papers included in the systematic review and their quality assessment score

Author (year)	Country	Study design	Total sample *n* (MBI *n*)	Sample composition	Outcome measure(s) of interest	MBI and control group	Main findings relating to PLE and/or mindfulness	Quality assessment score
Burke et al. (2020)	USA	Quasi-experimental	60	University students with at least one risk factor (mild to moderate depression and/or PLEs)	PDI (Peters et al., 2024) FFMQ (Baer et al., 2006)	Resilience training intervention 4 weeks of 1.5-h sessions Group format Encouragement of home practice No control group	1. PLEs were significantly negatively correlated with mindfulness (*p* < .05). 2. Significantly lower levels of PLEs, following the intervention compared to baseline (*p* < .001). 3. No significant increase in mindfulness skill observed from baseline to post-intervention (*p* = .18).	0.95 (strong)
DeTore et al. (2023)	USA	RCT	107 (54)	University students with at least one risk factor (mild to moderate depression and/or PLEs.	PDI (Peters et al., 2024) FFMQ (Baer et al., 2006)	Based on resilience training intervention (Burke et al., 2020) 4 weeks of 1.5-h sessions Group format Encouragement of home practice Waitlist control group	1. Significant time × group interaction for the PLEs (F(1,86) = 7.66, *p* = 0.007, *η_p_* ^2^ = 0.08). 2. MBI group showed a greater decrease in PLEs (*t*(86) = −2.77, *p* = 0.007, *d* = −0.58) at post-intervention compared to the waitlist group. 3. Significant time × group interaction for mindfulness (F(1,46) = 8.32, *p* = 0.006, *η_p_* ^2^ = 0.15). 4. The MBI group showed a greater increase in mindfulness (*t*(46) = 2.89, *p* = 0.006, *d* = 0.79) at post-intervention compared to the waitlist group.	0.85 (strong)
Hosseini et al. (2021)	Iran	Cross-sectional	168	University students	RHS Persian version (Goodarzi, 2009) FFMQ Persian version (Ahmadvand, Heydarinasab, & Shairi, 2013)	N/A	Significant relationship between hallucination proneness and each of the 5 facets of the FFMQ. Positive correlation between observing score and hallucination (Observing *r* = .22), but negative correlation for all of the other facets (Describing *r* = −.24; Acting *r* = −.31; Not judging *r* = −.20; Not reacting *r* = −.17)	0.70 (good)
Kingston, Lassman, Matias, and Ellett (2019) Study 1	UK and online	Cross-sectional	410	University students and the general population	Paranoia Scale (Fenigstein & Vanable, 1992) FFMQ (Baer et al., 2006)	N/A	^a^ Mindfulness negatively correlated with PLEs (*r* = −.57, *p* < .001).	0.95 (strong)
Kingston et al. (2019) Study 3	UK	RCT	68 (34)	University students	PDS Paranoia Scale (Bodner & Mikulincer, 1998) FFMQ (Baer et al., 2006)	Based on guided mindfulness meditation (Chadwick, 2006) 1 week of 10-min daily practice Audio track of guided meditation practice No additional home practice Active control - Guided visual imagery	1. Time × condition interaction was not significant for PLEs (F(1, 66) = 1.19, *p* = .29). 2. Main effect of time: PLEs significantly reduced pre-post intervention (F(1, 66) = 42.00, *p* < .001). 3. Main effect of group: No difference between the MBI and control groups (F(1, 68) = 1.29, *p* = .26).	0.88 (strong)
Langer, Cangas, and Gallego (2010)	Spain	RCT	38 (18)	University students with at least one risk factor (distressing and anxiety-provoking hallucination-like experiences)	RHS Spanish version (Cangas, Langer, & Moriana, 2011)	Based on MBCT (Segal et al., 2002) 8 weeks of 1-h sessions Group format Encouragement of home practice Active control - Watching videos on sociopolitical topics and engaging in a group discussion	No significant reduction in PLEs in the experimental group compared to the active control group (*p* = .78, *d* = .08).	0.50 (adequate)
Liu (2019)	China	Quasi-experimental	81	University students	SCL–90 R Paranoid Ideation Chinese version (Jin, Wu, & Zhang, 1986)	Based on MBCT (Segal et al., 2002) 8 weeks of 2.5-h sessions Group format Encouragement of home practice No control group	MBI decreased PLEs (F = 19.857, *p* < .01).	0.85 (strong)
Lynn, McDonald, Sleight, and Mattson (2023)	USA	Cross-sectional	527	University students	LSHS-R (Bentall & Slade, 1985) FFMQ (Baer et al., 2006)	N/A	^b^ A significant, negative correlation was found between total FFMQ and PLEs (*r* = −.28, *p* < .001).	1.00 (strong)
McDonald, Valmaggia, Antonova, and Chadwick (2024)	UK and online	RCT	24 (12)	Combined university students and the general population with at least one risk factor (high schizotypy)	Paranoia Scale (Fenigstein & Vanable, 1992)	Use of the Headspace app 10 days of 10-min daily practice Audio track of guided meditation practice No additional home practice Active control –Reflective journaling	No overall group effect was observed from baseline to post-intervention for PLEs.	0.92 (strong)
Moran et al. (2021)	Ireland	Cross-sectional	77	University students and the general population	LSHS-R (Bentall & Slade, 1985) MAAS (Brown & Ryan, 2003)	N/A	^a^ Mindfulness was negatively correlated with PLEs (*r* = −.38, *p* = .001).	1.00 (strong)
Muneghina et al. (2021)	UK and online	RCT	72 (37)	General population	Paranoia Scale (Fenigstein & Vanable, 1992) MAAS (Brown & Ryan, 2003)	5 days of 10-minute daily practice Audio track of guided meditation practice No additional home practice Waitlist control group	1. Significant condition × time interaction on PLEs (F(1.60, 111.91) = 12.09, *p* < 0.001). 2. Main effect of condition on PLEs: Significant difference in PLEs scores between intervention and control groups (F(1, 70) = 994.713, *p* < 0.001). 3. PLEs were significantly lower in the MBI compared with the control group at post-intervention (*p* < 0.001), but not at baseline (*p* = 0.122). 4. No significant condition × time interaction on mindfulness scores (F(1.693, 118.496) = 1.537, *p* = 0.221). 5. Main effect of condition on mindfulness: No significant difference in scores between intervention and control groups (F(1, 70) = 0.002, *p* = 0.966).	0.77 (good)
Oliver et al. (2012)	UK and NZ	Cross-sectional	700	University students	PDI (Peters et al., 2024) KIMS (Baer et al., 2004)	N/A	^a^ Significant inverse correlation between mindfulness and PLEs (*r* = −.19, *p* < .001).	0.90 (strong)
Pagnini et al. (2018)	Italy	Cross-sectional	248	University students	SCL–90 R Paranoid Ideation Italian version (Sarno, Preti, Prunas, & Madeddu, 2011) LMS (Pirson et al., 2012) Italian translation	N/A	A negative, nonsignificant correlation was found between mindfulness and the PLEs (*r* = −.086, *p* = .19).	0.75 (good)
Palacios-García et al. (2018)	Spain	Cross-sectional	526	University students	PDI Spanish version (López-Ilundain, Pérez-Nievas, Otero, & Mata, 2006) LSHS-R Spanish version (Fonseca-Pedrero et al., 2010) MAAS Spanish version (Soler et al., 2012)	N/A	Significant inverse correlations were found between mindfulness scores and the overall scores of the PDI–21 (*r* = −.470, *p* < .001), and the LSHS-R (*r* = −.525, *p* < .001).	0.95 (strong)
Perona-Garcelán et al. (2014)	Spain	Cross-sectional	83	University students with at least one risk factor (high or low hallucination proneness)	LSHS-R Spanish version (Fonseca-Pedrero et al., 2010) SMQ (Chadwick et al., 2008) Spanish translation	N/A	Subjects with high proneness showed significantly lower levels on the mindfulness scale compared to participants with low proneness (*t*(81) = −4.56, *p* < .001; *d* = 1.12).	0.90 (strong)
Peters et al. (2016)	UK	Cross-sectional	92	General population	SAPS (Andreasen, 1984) AANEX (Brett et al., 2007) SMQ (Chadwick et al., 2008)	N/A	^a^ Total lifetime AANEX was not significantly correlated to total mindfulness (*r* = .031, *p* = .78). Current AANEX was not significantly correlated to total mindfulness (*r* = .079, *p* = .47). Total positive symptoms were not significantly correlated to total mindfulness (*r* = .092, *p* = .41).	0.90 (strong)
Shore et al. (2018)	UK and online	RCT	60 (29)	University students and the general population	Paranoia Scale (Fenigstein & Vanable, 1992) FFMQ (Baer et al., 2006)	Based on guided mindfulness meditation (Chadwick, 2006) 2 weeks of 10-min daily practice Audio track of guided meditation practice plus access to ‘Learning Meditation Online’ website Encouragement of home practice Waitlist control group	1. Significant group × time interaction on PLEs (F(1.70, 98.72) = 5.70, *p* = .01). 2. Significant difference in PLEs between MBI and waitlist control at post-intervention (*t*(69.9) = 2.32, *p* = .024, *d* = 0.74 95% CI for *d* = (0.22, 1.27)). 3. MBI group showed a significant decrease in PLEs over time pre- to post-intervention (*t*(33) = 4.18, *p* < .001, *d* = 0.60, 95% CI for *d* = (0.11, 1.08)). ^a^4. Increase in mindfulness score at post-intervention for MBI compared to control (*p* = .001, *d* = −.89).	0.88 (strong)
Torok and Keri (2022)	Hungary	Cross-sectional	300	General population	sO-LIFE Unusual Experiences subscale Hungarian version (Kocsis-Bogár, Nemes, & Perczel-Forintos, 2016) MAAS Hungarian version (Simor, Petke, & Köteles, 2013)	N/A	Mindfulness significantly predicted PLEs (*R* ^2^ = 0.06, *β* = −0.22, SE = 0.06, *p* < 0.01).	0.95 (strong)

*Note*: USA, United States of America; UK, United Kingdom; NZ, New Zealand; RCT, randomized controlled trial; PLEs, psychotic-like experiences; PDI, Peters Delusions Inventory; RHS, Revised Hallucination Scale; PDS, Paranoia and Depression Scale; SCL-90 R, Symptom Checklist 90 Revised; LSHS-R, Launay Slade Hallucination Scale-Revised; SAPS, Scale for the Assessment of Positive Symptoms; AANEX, Appraisals of Anomalous Experiences Interview; sO-LIFE, short version of the Oxford-Liverpool Inventory of Feelings and Experiences; FFMQ, Five Facets of Mindfulness Questionnaire; MAAS, Mindful Attention Awareness Scale; KIMS, Kentucky Inventory of Mindfulness Skills; LMS, Langer Mindfulness Scale; SMQ, Southampton Mindfulness Questionnaire.

a Results provided by corresponding author.

b Results from Supplementary Material.

### Characteristics of studies included

The total number of participants included across all studies was 3,641. The studies were conducted in 11 different countries, with some papers having multiple recruitment sites or online recruitment: United Kingdom (*k* = 7), Spain (*k* = 3), United States (*k* = 3), and one study each from China, Hungary, Iran, Ireland, Italy, and New Zealand. The systematic review included studies with cross-sectional (*k* = 10), RCT (*k* = 6), and quasi-experimental (*k* = 2) designs, with one paper contributing a cross-sectional study and an RCT.

### Characteristics of participants

Only one study reported no demographic information about the participants (Hosseini et al., 2021). Of the remaining studies, 14 reported more female participants (ranging from 53.2 to 89.2% female), and 2 reported more male participants (43.1 and 49.3% female). Additionally, of the 16 studies that reported participant characteristics, there was an age range of 18–80 years old, with a pooled mean age of 24.18 years and a pooled standard deviation of 9.03. All 17 studies reported some information on their sample composition: University students (*k* = 6), general population (*k* = 3), combined university students and general population (*k* = 3), university students who were reporting at least one risk factor, such as reporting some current level of PLE or mild depression (*k* = 4), and combined university students and general population who were reporting at least one risk factor, such as high schizotypy (*k* = 1).

### PLE measures

All studies had at least one measure of PLEs. Fifteen studies collected data for individual PLEs when only one symptom was focused on; 1 study focused on general PLEs when the focus was on a collective group of symptoms; and 2 studies used multiple measures to assess PLEs. Paranoia was explored in seven of the studies, four of which used the Paranoia Scale (Fenigstein & Vanable, 1992), two used the Paranoia Ideation subscale of the Symptom Checklist 90-Revised (Derogatis & Unger, 2010), and one used the Paranoia Scale of the Paranoia and Depression Scale (Bodner & Mikulincer, 1998). Hallucinations were investigated in six of the studies, four of which used the Launay Slade Hallucination Scale-Revised (Bentall & Slade, 1985), and two used the Revised Hallucination Scale (Morrison, Wells, & Nothard, 2002). Four papers explored delusions, and all used the Peters Delusions Inventory (Peters et al., 2004). One study investigated unusual experiences using the Unusual Experiences subscale of the short version of the Oxford-Liverpool Inventory of Feelings and Experiences (Mason, Linney, & Claridge, 2005). One study explored positive symptoms generally, using the Scale for the Assessment of Positive Symptoms (Andreasen, 1984). Finally, one study investigated anomalous experiences using the Appraisals of Anomalous Experiences Interview (Brett et al., 2007). While some studies used validated translated versions of these measures, others used translations that were created specifically for their study without prior validation (see Table 1).

### Mindfulness measures

Three papers collected no data on mindfulness, but the remaining 14 papers used a mindfulness measure: 6 studies used the Five Facets of Mindfulness Questionnaire (FFMQ; Baer et al., 2006), 4 studies used the Mindful Attention Awareness Scale (Brown & Ryan, 2003), 2 studies used the Southampton Mindfulness Questionnaire (Chadwick et al., 2008), 1 study used the Kentucky Inventory of Mindfulness Skills (Baer, Smith, & Allen, 2004), and 1 study used the Langer Mindfulness Scale (Pirson, Langer, Bodner, & Zilcha-Mano, 2012). Some studies used translated versions of the measures, see Table 1.

### Mindfulness-based interventions

Eight of the studies included an MBI, four used a face-to-face group format, and four studies used online audio tracks of guided mindfulness practice. The amount of mindfulness practice undertaken ranged from 50 min (10 min daily for 5 consecutive days) to 20 h (8 weeks of 2.5-h sessions). Five interventions encouraged home practice, whereas two did not. Two of these studies were quasi-experimental and, therefore, had no control group, reporting within-subjects results only. For the other six studies, three had waitlist control groups, and three had active controls (reflective journalling, *k* = 1; audio track of guided visual imagery, *k* = 1; watching videos on sociopolitical topics and engaging in a group discussion, *k* = 1) (see Table 1).

### Main findings

#### Relationship between PLEs and mindfulness

Ten cross-sectional studies examined the relationship between PLEs and mindfulness, and one quasi-experimental study reported the baseline association between the two measures. Of these 11 studies, five studies focused on hallucinations, 4 on delusions, 2 on paranoia, 1 on unusual experiences, 1 on positive symptoms, and 1 on current and lifetime anomalous experiences. Two studies reported on more than one PLE.

Ten studies reported at least one Pearson’s correlation coefficient for total PLE scores and either the total mindfulness scores or subscale scores. Eight studies found significant negative correlations (range *r* = −.38 to −.57), indicating that greater mindfulness skills were associated with lower scores on the PLE measures, with small-medium effect sizes. One study found a positive correlation between the observing facet of the FFMQ and hallucination proneness (*r* = .22), suggesting higher levels of observation were associated with increased proneness to hallucination (Hosseini et al., 2021), and two further studies reported nonsignificant correlations between PLEs and mindfulness (Pagnini et al., 2018; Peters et al., 2016). Finally, one cross-sectional study by Perona-Garcelán et al. (2014) found that participants categorized as having ‘high hallucination proneness’ scored significantly lower on the mindfulness scale compared to participants categorized as ‘low hallucination proneness’ (Cohen’s *d* = 1.12).

Studies were included in the meta-regression if a correlation coefficient between the overall PLE score and the overall mindfulness score was reported. Of the 11 studies identified above, 8 were included in the meta-regression (see Table 2). Additionally, one study contributed 2 correlations (Palacios-García et al., 2018) and another study contributed 3 correlations (Peters et al., 2016), resulting in a total of 12 correlations included in the meta-regression.

**Table 2.**
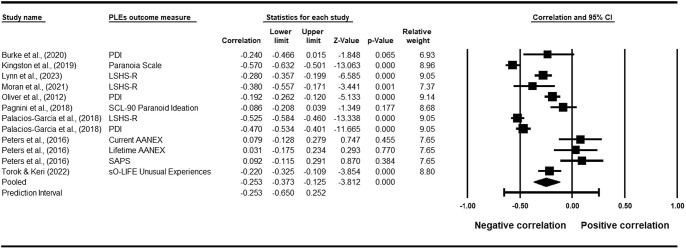
Meta-regression and Forest Plot of the relationship between mindfulness and PLEs (seven cross-sectional studies; one RCT, one quasi-experimental design)

The meta-regression found a pooled correlation of *r =* −0.25 (95% confidence intervals [CIs]: −0.37 to −0.13, *p* < .001), indicating a small negative association between PLEs and mindfulness. This suggests that higher levels of mindfulness are associated with lower levels of PLEs. The *Q*(11) = 165.86 (*p* < .001) and the *I* ^2^ statistic was 93%, suggesting 93% of the variability in effect sizes reflected true heterogeneity rather than sampling error. Given the significant *Q*-value and the *I* ^2^ value exceeded 50%, substantial heterogeneity was deemed to be present. Additionally, visual inspection of the Funnel Plot revealed that 8 out of 12 studies fell outside of the funnel, and Egger’s regression intercept (*B*_0_) was 4.26 (95% CI: −1.93 to 10.44; *t*(10) = 1.53; one-tailed *p*-value = .078). The Funnel Plot and Egger’s test may indicate the presence of publication bias. Taken together, these findings indicate a small negative association between mindfulness and PLEs. However, due to the high levels of heterogeneity and potential publication bias, the results of this meta-regression should be interpreted with caution.

### Effect of MBIs on PLEs

Two quasi-experimental studies and six RCTs investigated the impact of MBIs on PLEs. Of these, five studies focused on paranoia, two on delusions, and one on hallucinations. Five studies found the MBIs significantly reduced PLEs with medium effect sizes (range *d* = .58, .74). One study found that participation in both the MBI and the guided visual imagery active control reduced PLEs but the MBI was not superior to the active control (*d* = −.18, 95% CI: −.65 to .30). Additionally, two studies found no reduction of PLEs following MBI completion and no differences between the MBI group compared to the active control groups of reflective journalling or watching videos on sociopolitical topics and engaging in a group discussion.

Quasi-experimental studies were not included in the meta-analysis. Of the six RCTs identified above, five were included in the meta-analysis, and one was excluded as data were presented as change scores (see Table 3). The meta-analysis found a pooled standard mean difference of 0.09 (95% CI: −0.61 to 0.79, *p* = 0.80), indicating a nonsignificant effect of MBI’s on PLEs. The *Q*(4) = 35.98 (*p* < 0.001) and the *I* ^2^ statistic was 89%, suggesting 89% of the variability in effect sizes reflected true heterogeneity rather than sampling error. Given the significant *Q*-value and the *I* ^2^ value exceeded 50%, substantial heterogeneity was deemed present in the meta-analysis. Additionally, visual inspection of the Funnel Plot revealed that two out of the five studies fell outside of the funnel, and Egger’s regression intercept (*B*_0_) was −8.30 (95% CI: −19.86 to 3.26; *t*(3) = 2.28; one-tailed *p*-value = 0.05). The Funnel Plot and Egger’s test may indicate the presence of publication bias, although the Egger’s test had low power as there were fewer than 10 studies (Page et al., 2024). Taken together, these results suggest that MBI participation does not reduce PLEs. However, due to high levels of heterogeneity and potential publication bias, the results of this meta-analysis should be interpreted with caution.

**Table 3.**
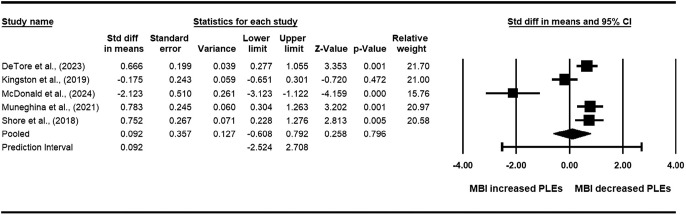
Meta-analysis and Forest Plot of RCTs examining the effect of MBIs on PLEs

### Quality analysis results

Quality analysis found that 14 studies were ‘strong’, three were ‘good’, and one was ‘adequate’ (see Table 1), indicating the robustness of the literature overall.

## Discussion

This systematic review and the associated meta-analyses provide the first synthesis of the literature, including 17 studies exploring mindfulness and PLEs. Two preregistered research questions were addressed by examining the relationship between mindfulness and PLEs (*k* = 11) and assessing the effectiveness of MBIs for individuals experiencing PLEs in nonclinical populations (*k* = 8). Most papers were assessed as having either a strong or good quality rating (*k* = 16), with only one paper in the adequate category, suggesting that the body of literature overall is robust. Overall, two key findings emerged from the review. First, mindfulness and PLEs were negatively correlated in the meta-regression, finding a small association. This association was consistent across a number of studies, despite the use of different PLEs and mindfulness measures, suggesting that higher levels of mindfulness are associated with lower scores on PLE measures. Second, there was no effect of MBIs on PLEs as the summary effect in the meta-analysis was not significant. It should be noted that all meta-analyses undertaken resulted in high levels of heterogeneity, with evidence of some publication bias; therefore, all findings need to be interpreted cautiously.

Collectively, these findings suggest that PLEs and mindfulness are negatively correlated and there was no evidence for the effectiveness of MBIs in reducing PLEs in nonclinical populations. It is important to consider possible explanations for this null effect. Some MBIs may not be sufficient in duration or intensity to produce measurable change in such experiences in these populations. Additionally, the variability in intervention content and delivery, ranging from brief daily audio sessions to intensive multi-week programs, may have contributed to inconsistent outcomes. In some cases, active control conditions were also found to reduce PLEs, suggesting that nonspecific therapeutic factors, such as structured self-reflection or relaxation, may partially account for improvements. Furthermore, baseline severity of PLEs may impact the effectiveness of MBIs, although this was not controlled for in any of the studies and should therefore be addressed in future research. Many studies recruited from universities or the general population with low levels of PLEs, potentially resulting in floor effects that could have limited the degree of observable change. Finally, some of the measures may not have been analyzed using disaggregated data between different dimensions of unusual experiences (e.g. the Peters Delusions Inventory has dimensions of frequency, distress, and interference for each item). We might expect that mindfulness would reduce distress and interference for PLEs, but not necessarily frequency or presence/absence, and analyzing the measure as a total score could obscure this.

The review raises some important methodological and conceptual challenges. We note the inherent challenge of attempting to quantify subjective internal states using objective research tools. This difficulty is not unique to psychiatric research, but is perhaps particularly relevant in mindfulness studies, where the construct itself is variably defined and its practices differ substantially across interventions. To improve methodological rigor, future research would benefit from (i) more detailed and standardized reporting of mindfulness practices and delivery (e.g. length, intensity, and instructor training); (ii) multi-method assessment strategies that complement self-report with behavioral, physiological, or observational measures, and (iii) the use of longitudinal or experience-sampling designs to capture dynamic, real-time fluctuations in mindfulness practice and related subjective states, thereby reducing retrospective bias and enhancing ecological validity. An additional consideration concerns the heterogeneity of interventions classified as ‘mindfulness-based’. We note that intervention protocols differ in the amount of mindfulness undertaken, and mindfulness may be just one component of an intervention that includes other strategies. Considering MBIs collectively has limitations because the mechanisms driving outcomes, and the relative contribution of mindfulness specifically, may differ between interventions where mindfulness is a component vs the central therapeutic element. Future research might usefully differentiate MBIs where mindfulness is a component versus the central therapeutic element and assess whether clinical outcomes correlate with the amount of mindfulness practice undertaken.

This systematic review had several strengths, including adherence to PRISMA guidelines, preregistration of the protocol, and the use of both narrative synthesis and meta-analysis approaches. A comprehensive and systematic search strategy was applied across multiple databases, with rigorous screening and quality assessment processes. Nevertheless, several limitations should also be acknowledged. First, many studies included university students who are not typical of the general population, which may limit generalizability, and several studies lacked comprehensive demographic reporting, which limited the ability to evaluate the potential influence of factors such as age, gender, and cultural context on the findings. In cases where demographic information or sample characteristics were reported, participants were predominantly drawn from Western, Educated, Industrialized, Rich, and Democratic (WEIRD) populations (Henrich, Heine, & Norenzayan, 2010). This overrepresentation of WEIRD samples highlighted a notable limitation in the generalizability of the results, as such populations represented only a small proportion of global human diversity and are not representative of the global majority. Therefore, future research might usefully examine the effect of MBIs on individuals from low- and middle-income countries and include samples from a range of ethnic backgrounds. Second, the high heterogeneity observed in all meta-analyses is reflective of the considerable variation in study methodologies, measures, and populations, making it difficult to draw well-substantiated conclusions. Third, all studies included relatively small follow-up periods, and therefore it is not possible to determine the durability of any effects found and whether individuals continue to practice and potentially benefit from mindfulness. Fourth, the data were not double-extracted by an independent researcher. Fifth, autism spectrum disorder (ASD) was not specified as an exclusion criterion in this review. Although some included studies incorporated measures such as schizotypy, none recruited or reported ASD samples. We, therefore, included studies if they met the predefined criteria, but we recognize that not explicitly addressing ASD represents a limitation, given the potential overlap between ASD features and PLEs. Finally, given that the presence of PLEs has been associated with an increased risk of developing psychotic disorders, particularly when PLEs are persistent and reoccurring (Dominguez et al., 2009), there is a notable absence of data in the literature about whether MBIs might prevent transition to psychosis or an at-risk mental state in nonclinical populations, and future research might also usefully measure a broader range of clinically-relevant outcomes.

In conclusion, this systematic review and the associated meta-analyses provide evidence that higher levels of mindfulness may be associated with fewer PLEs. However, there was no evidence for the effectiveness of MBIs in reducing PLEs in nonclincial populations. Findings need to be interpreted cautiously given the relatively small number of studies and the high heterogeneity in the meta-analyses undertaken. Future studies are needed to determine whether MBIs might prevent the transition to psychosis or an at-risk mental state and might usefully measure a broader range of clinically relevant outcomes.

## Data Availability

The data that support the findings of this review are available from the corresponding author upon reasonable request.
